# Author Correction: Tracking SARS-COV-2 variants using Nanopore sequencing in Ukraine in 2021

**DOI:** 10.1038/s41598-023-29749-9

**Published:** 2023-02-13

**Authors:** Anna Yakovleva, Ganna Kovalenko, Matthew Redlinger, Mariia G. Liulchuk, Eric Bortz, Viktoria I. Zadorozhna, Alla M. Scherbinska, Joel O. Wertheim, Ian Goodfellow, Luke Meredith, Tetyana I. Vasylyeva

**Affiliations:** 1 Medical Sciences Division, University of Oxford, Oxford, UK; 2 Division of Infectious Diseases and Global Public Health, University of California San Diego, San Diego, CA USA; 3 Division of Virology, Department of Pathology, University of Cambridge, Cambridge, UK; 4 Department of Biological Sciences, University of Alaska Anchorage, Anchorage, AK USA; 5 State Institution “L.V. Hromashevskyi Institute of Epidemiology and Infectious Diseases of the National Academy of Medical Sciences of Ukraine”, Kyiv, Ukraine

Correction to: *Scientific Reports* 10.1038/s41598-022-19414-y, published online 21 September 2022

The Acknowledgements section in the original version of this Article was incomplete.

“We would like to thank Oxford Nanopore for providing funding support for travel to Ukraine. We are grateful to all authors from the originating laboratories responsible for obtaining the specimens, as well as the submitting laboratories where the genome data were generated and shared via GISAID, on which this research is based (Supplementary Table S4).”

now reads:

“We would like to thank Oxford Nanopore for providing funding support for travel to Ukraine. We are grateful to all authors from the originating laboratories responsible for obtaining the specimens, as well as the submitting laboratories where the genome data were generated and shared via GISAID, on which this research is based (Supplementary Table S4). We would like to acknowledge support from the AIDS Healthcare Foundation."

The original Article has been corrected.

